# Serum C-reactive protein to albumin ratio as a reliable marker of diabetic neuropathy in type 2 diabetes mellitus

**DOI:** 10.17305/bb.2024.10426

**Published:** 2024-10-01

**Authors:** Gulali Aktas

**Affiliations:** 1 Department of Internal Medicine, Abant Izzet Baysal University Hospital, Bolu, Turkey

**Keywords:** Type 2 diabetes mellitus (T2DM), diabetic neuropathy (DN), inflammation, C-reactive protein to albumin ratio (CAR)

## Abstract

In various diseases characterized by inflammation, the C-reactive protein to albumin ratio (CAR) serves as a marker of inflammation. Type 2 diabetes mellitus (T2DM) is frequently complicated by diabetic neuropathy (DN) and timely diagnosis is crucial for treatment and potential reversal of this complication. Since both DN and T2DM are associated with chronic, low-grade inflammation, our study aimed to evaluate CAR levels in type 2 diabetic subjects with DN and compare them to those in subjects without DN. Patients presenting to our institutional outpatient clinics were divided into two groups based on the presence of DN. Data on characteristics and laboratory measures, including CAR, were compared between the DN and non-DN groups. The median CAR in the DN and non-DN groups was 2.19% (range 0.2%–49%) and 0.56% (range 0.02%–5.8%), respectively (*P* < 0.001). CAR showed significant positive correlations with weight (*r* ═ 0.19, *P* ═ 0.01), body mass index (BMI) (*r* ═ 0.11, *P* ═ 0.03), waist circumference (*r* ═ 0.10, *P* ═ 0.046), fasting glucose (*r* ═ 0.14, *P* ═ 0.004), serum creatinine (*r* ═ 0.25, *P* < 0.001), triglyceride (*r* ═ 0.17, *P* < 0.001), and LDL-cholesterol (*r* ═ 0.13, *P* ═ 0.001) levels, and an inverse correlation with estimated glomerular filtration rate (eGFR) (r ═ −0.16, *P* <0.001). Additionally, CAR demonstrated a sensitivity of 78% and specificity of 73% for predicting DN at a threshold of 1.02% (area under curve [AUC] 0.84, 95% confidence interval [CI] 0.82–0.87, *P* < 0.001). High CAR levels were independently associated with an increased risk of DN (odds ratio [OR] 1.34, 95% CI 1.08–1.62, *P* < 0.001). Elevated CAR levels may thus be considered a potential marker for DN in T2DM patients.

## Introduction

Type 2 diabetes mellitus (T2DM) is associated with insulin resistance and chronic hyperglycemia. Injury to major organs and systems, including cardiovascular diseases, diabetic kidney injury, diabetic foot ulcers, diabetic retinopathy, and diabetic neuropathy (DN), may occur when hyperglycemia persists in diabetic subjects [1]. Unfortunately, most diabetic patients have at least one of these macrovascular or microvascular complications of the disease, and among these, cardiovascular events are the leading cause of mortality in the diabetic population [2].

DN is one of the most common complications of diabetes that can lead to pain, loss of sensation, and limb amputation, impairing the quality of life [3]. DN has a complex pathophysiology and affects both components (autonomic and somatic) of the nervous system. Neuropathic pain, tingling, paraesthesia, numbness, and a burning feeling are characteristic symptoms of DN. These symptoms mainly occur in toes, feet, or legs. Inflammation in the nerve cells is triggered by advanced glycosylation end products via activation of nuclear factor kappa beta, interleukins, nitric oxide synthase, and tumor necrosis factor-alpha (TNF-α) [4]. In DN, this inflammation at the cellular level causes other inflammatory cells to come to the area, secreting inflammatory cytokines that turn the environment into a site of inflammation. Ischemia and hypoxia develop within the nerve cells, resulting in mitochondrial dysfunction and excessive production of reactive oxygen species [5].

These accumulating pieces of evidence suggest that diabetic peripheral neuropathy is associated with an increased burden of inflammation. On the other hand, a novel inflammatory marker, the C-reactive protein to serum albumin ratio (CAR), has been introduced as a new and promising marker of inflammatory conditions and as a diagnostic and prognostic marker of various diseases. For example, the CARE TIME study showed that elevated levels of CAR could be a marker of diabetic nephropathy in T2DM [6]. Moreover, CAR is considered a diagnostic marker in colorectal carcinoma [7], and pancreas malignancy [8], and a prognostic marker in sepsis [9]. All of these conditions are associated with inflammation, as DN is. However, to the best of our knowledge, CAR levels have not been studied in T2DM patients with DN.

In the present study, we aimed to observe CAR levels in DN subjects and compare them to those in diabetic patients without DN.

## Materials and methods

Patients with T2DM who were treated in the outpatient internal medicine clinics of our institution were enrolled in the present retrospective cross-sectional analysis. Patients with active infections, inflammatory diseases, advanced liver disease, cancer, and pregnancy were excluded from the study. Subjects receiving anti-inflammatory drugs were also excluded. The study population was divided into two groups based on the presence of DN: diabetic patients with DN (DN group) and diabetic subjects without DN (non-DN group). The diagnosis of DN was based on the recommendations of the Toronto Diabetic Neuropathy Expert Group [10].

Age, sex, body weight, height, duration of T2DM, systolic and diastolic blood pressure, smoking, and drinking habits of the study cohort were recorded from the institutional computerized database and from the patients’ files. The presence of diabetic nephropathy and retinopathy was also recorded. Body mass index (BMI) was calculated by dividing weight (kg) by the square of height (m^2^).

Laboratory parameters, including blood leukocyte count, hemoglobin, hematocrit, platelet count, fasting glucose, glycated hemoglobin (HbA1c), blood urea, creatinine, estimated glomerular filtration rate (eGFR), serum uric acid, liver function tests (aspartate [AST] and alanine [ALT] transaminases), total LDL, and HDL cholesterol levels, plasma triglyceride, C-reactive protein (CRP), and albumin, were also obtained and recorded. All biochemical assays were conducted with an automatic analyzer (Architect c 8000, Abbott Laboratories, Chicago, USA) following the manufacturer’s instructions. The CAR value was calculated by dividing CRP by serum albumin. General characteristics and laboratory parameters of the DN and non-DN groups were compared. We further analyzed the diabetic subjects with or without neuropathy after excluding subjects either with nephropathy or retinopathy.

### Ethical statement

The study was conducted in accordance with the Declaration of Helsinki and was approved by the Institutional Review Board (approval date: April 11, 2023, approval number: 2023/105).

### Statistical analysis

Statistical software (SPSS 16.0 for Windows, IBM Co., Chicago, IL, USA) was used for statistical analyses of the study variables. The distribution skewness of the study variables was assessed with the Kolmogorov–Smirnov test. Variables that fit a normal distribution were expressed as mean and standard deviation, while variables with skewed distribution were expressed as median (min–max). Categorical variables were expressed as number and percentage. Comparison of variables with a normal distribution was conducted with independent samples *t*-test, while the comparison of variables with skewed distribution was performed with the Mann–Whitney *U* test. The chi-square test was used to compare categorical variables among study groups. Correlations between CAR and other study variables were analyzed using Pearson’s correlation analysis test. The sensitivity and specificity of CAR in indicating DN were analyzed with ROC analysis. Logistic regression analysis (with independent variables: age, BMI, diabetes duration, fasting plasma glucose, HbA1c, serum creatinine, LDL-cholesterol, and CAR) was conducted to identify independent risk factors for DN. Statistical significance was considered when the *P* value was lower than 0.05.

## Results

The study cohort consisted of 697 diabetic individuals, with 266 in the DN group and 431 in the non-DN group. The mean age of the DN and non-DN groups was 60 ± 10 years and 56 ± 11 years, respectively. Age did not show a statistically significant difference between the study groups (*P* ═ 0.11). In the DN group, 172 (65%) of the subjects were women and 94 (35%) were men, while in the non-DN group, 219 (56%) were women and 212 (44%) were men. There was no statistically significant difference between the DN and non-DN groups in terms of gender (*P* ═ 0.06).

Body weight (*P* ═ 0.6), waist circumference (*P* ═ 0.56), systolic blood pressure (*P* ═ 0.17), and diastolic blood pressure (*P* ═ 0.07) did not show statistically significant differences between the DN and non-DN groups. However, the BMI of the DN group (31.1, range 17–46.3 kg/m^2^) was significantly higher than that of the non-DN group (30.8, range 19–55.4 kg/m^2^) (*P* ═ 0.02). Table 1 summarizes the general characteristics of the DN and non-DN groups and Table 2 shows the laboratory data of the DN and non-DN groups.

**Table 1:** General characteristics of the DN and non-DN groups

		**DN group**	**Non-DN group**	***P***
Sex, *n* (%)	Male	172 (65)	219 (56)	0.06
	Female	94 (35)	212 (44)	
Diabetic nephropathy, *n* (%)	Present	161 (60.5)	120 (28)	**<0.001**
	Absent	105 (39.5)	311 (72)	
Diabetic retinopathy, *n* (%)	Present	54 (20.3)	29 (6.7)	**<0.001**
	Absent	212 (79.7)	402 (93.3)	
		Mean ± standard deviation		
Age (years)		60 ± 10	56 ± 11	0.11
		Median (min.-max.)		
Weight (kg)		82 (48–120)	83 (46–150)	0.6
Waist circumference (cm)		105 (75–132)	105 (78–160)	0.56
BMI (kg/m^2^)		31.1 (17–46.3)	30.8 (19–55.4)	**0.02**
Diabetes duration (years)		6 (1–24)	3 (1–20)	**<0.001**
Systolic blood pressure (mmHg)		128 (90–180)	130 (90–200)	0.17
Diastolic blood pressure (mmHg)		75 (50–110)	80 (50–110)	0.07

Bold values indicate statistical significance. DN: Diabetic neuropathy; BMI: Body mass index.

**Table 2:** Laboratory parameters of the DN and non-DN groups

	**DN group**	**Non-DN group**	***P***
	Mean ± standard deviation		
Leukocyte count (k/mm^3^)	6.9 ± 2	6.8 ± 2.3	0.33
Hemoglobin (g/dL)	13.1 ± 1.6	13.5 ± 1.7	0.16
Hematocrit (%)	39 ± 4	40 ± 5	0.27
Platelet count (k/mm^3^)	262 ± 36	256 ± 34	0.35
Aspartate transaminase (U/L)	24 ± 9	28 ± 10	0.68
Alanine transaminase (U/L)	23 ± 11	22 ± 12	0.65
	Median (min.-max.)		
C-reactive protein (mg/L)	9.5 (1–250)	2.2 (0.1–26.1)	**<0.001**
Serum albumin (g/dL)	4.3 (2.8–5.3)	4.4 (2.9–5.6)	**0.01**
Fasting plasma glucose (mg/dL)	177 (66–565)	147 (65–514)	**<0.001**
HbA1c (%)	8.3 (4.9–16.5)	7.7 (5.1–17.2)	**0.001**
Blood urea (mg/dL)	34 (17–222)	32 (13–258)	0.14
Serum creatinine (mg/dL)	0.83 (0.59–3.41)	0.8 (0.39–3.93)	**0.049**
eGFR (%)	99.5 (15.3–185)	99.8 (15.8–181)	0.057
Serum uric acid (mg/dL)	5.6 (1.8–10.4)	5.5 (2.4–13.6)	0.07
Triglyceride (mg/dL)	153 (47–850)	151 (50–856)	0.36
Total cholesterol (mg/dL)	187 (52–318)	200 (50–378)	0.14
LDL-cholesterol (mg/dL)	132 (21–200)	114 (29–244)	**0.03**
HDL-cholesterol (mg/dL)	46 (13–87)	44 (17–92)	0.1
CAR	2.19 (0.2–49)	0.56 (0.02–5.8)	**<0.001**

Bold values indicate statistical significance. DN: Diabetic neuropathy; LDL: Low-density lipoprotein; HDL: High-density lipoprotein; CAR: C-reactive protein to albumin ratio; eGFR: Estimated glomerular filtration rate.

The median CAR of the DN and non-DN groups was 2.19 (range 0.2–49) and 0.56 (range 0.02–5.8), respectively (*P* < 0.001).

Smoking (*P* ═ 0.27) and drinking alcohol (*P* ═ 0.44) rates did not show significant differences between the DN and non-DN groups. However, the rates of diabetic retinopathy (*P* < 0.001) and diabetic nephropathy (*P* < 0.001) were higher in subjects in the DN group compared to the patients in the non-DN group (Table 1).

Correlation analysis showed that CAR was significantly and positively correlated with age (*r* ═ 0.15, *P* < 0.001), weight (*r* ═ 0.19, *P* ═ 0.01), BMI (*r* ═ 0.11, *P* ═ 0.03), waist circumference (*r* ═ 0.10, *P* ═ 0.046), fasting plasma glucose (*r* ═ 0.14, *P* ═ 0.004), serum creatinine (*r* ═ 0.25, *P* < 0.001), triglyceride (*r* ═ 0.17, *P* < 0.001), and LDL-cholesterol (*r* ═ 0.13, *P* ═ 0.001) levels. Moreover, CAR was inversely correlated with eGFR levels (*r* ═ –0.16, *P* < 0.001).

**Figure 1.**
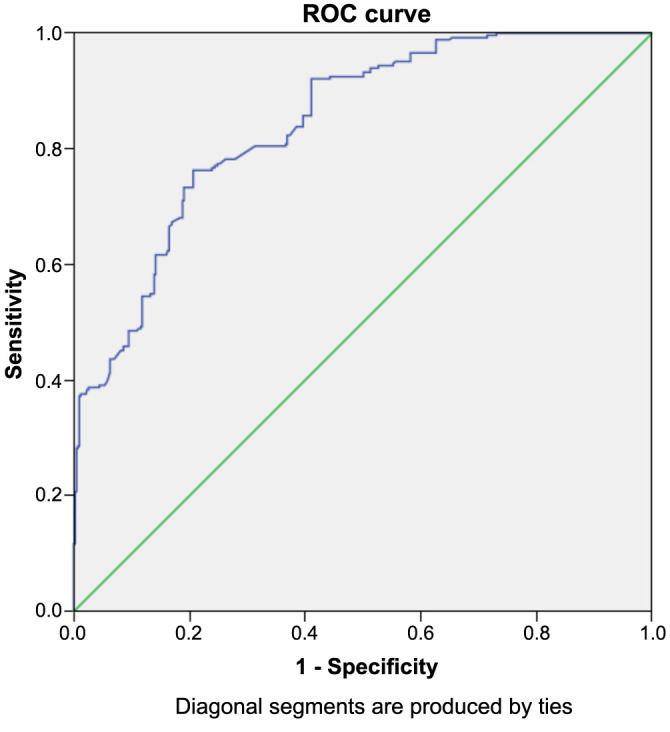
**ROC curve of CAR in detecting diabetic neuropathy.** ROC: Receiver operating characteristic curve; CAR: C-reactive protein to albumin ratio.

In ROC curve analysis, the sensitivity and specificity of CAR (when higher than 1.02%) in predicting DN were 78% and 73%, respectively (area under curve [AUC] 0.84, *P* < 0.001, 95% confidence interval [CI] 0.82–0.87). Figure 1 shows the ROC curve of CAR in detecting DN.

Logistic regression analysis was performed to determine whether CAR was an independent risk factor for DN (while also taking into account BMI, diabetes duration, fasting plasma glucose, HbA1c, serum creatinine, and LDL-cholesterol levels). A high CAR level was found to be an independent risk factor for DN (OR 1.34, *P* < 0.001, 95% CI 1.08–1.62).

In subgroup analysis, patients with either diabetic nephropathy or retinopathy were excluded, and the remaining 372 patients were included in the analyses (77 in the DN group vs 295 in without DN group). CAR was significantly increased in diabetic patients with DN compared to those without DN (*P* < 0.001). Table 3 shows data of the patients with and without DN after excluding patients with diabetic nephropathy and retinopathy.

**Table 3:** Laboratory parameters of the DN and non-DN patients in the subgroup cohort

		**DN group**	**Non-DN group**	***P***
Gender, *n* (%)	Women	56 (73)	151 (51)	**0.001**
	Men	21 (27)	144 (49)	
	Absent	212 (79.7)	402 (93.3)	
		Median (min.-max.)		
Age (years)		62 (47–76)	56 (29–81)	**<0.001**
Weight (kg)		86 (68–117)	85 (58–123)	0.33
Waist circumference (cm)		114 (88–132)	106 (82–137)	**<0.001**
BMI (kg/m^2^)		35.7 (24–46.3)	30.8 (21–46)	**<0.001**
Diabetes duration (years)		7 (1–21)	3 (1–20)	**<0.001**
Leukocyte count (k/mm^3^)		6.6 (3.7–9.7)	6.5 (3.7–9.9)	0.57
Hemoglobin (g/dL)		13.7 (12.5–15.9)	13.4 (12.2–17)	0.96
Hematocrit (%)		39 (36–47)	40 (35–51)	0.24
Platelet count (k/mm^3^)		246 (168–479)	232 (138–423)	0.83
C-reactive protein (mg/L)		12 (1–67)	2.7 (0.1–19)	**<0.001**
Serum albumin (g/dL)		4.4 (3.1–5)	4.4 (3.3–5.6)	0.15
Fasting plasma glucose (mg/dL)		136 (100–459)	143 (70–514)	0.68
HbA1c (%)		7.1 (6.3–12.8)	7.4 (5.1–15.9)	0.23
Blood urea (mg/dL)		32 (17–167)	36 (23–198)	0.23
Serum creatinine (mg/dL)		0.8 (0.7–1.2)	0.79 (0.4–0.9)	0.43
eGFR (%)		103 (35–125)	109 (44–145)	0.44
Triglyceride (mg/dL)		153 (77–850)	160 (50–856)	0.72
Total cholesterol (mg/dL)		162 (131–200)	202 (50–244)	**0.02**
LDL-cholesterol (mg/dL)		132 (21–200)	114 (29–244)	**<0.001**
HDL-cholesterol (mg/dL)		46 (13–87)	44 (17–92)	0.12
Aspartate transaminase (U/L)		19 (7–58)	19 (8–53)	0.32
Alanine transaminase (U/L)		16 (6–43)	20 (6–64)	0.15
CAR		2.7 (0.2–14.6)	0.6 (0.02–4.4)	**<0.001**

Bold values indicate statistical significance. DN: Diabetic neuropathy; BMI: Body mass index; LDL: Low-density lipoprotein; HDL: High-density lipoprotein; CAR: C-reactive protein to albumin ratio; eGFR: Estimated glomerular filtration rate.

In subgroup analysis, CAR was correlated with age (*r* ═ 0.12, *P* ═ 0.02), diabetes duration (*r* ═ 0.31, *P* < 0.001), fasting glucose (*r* ═ 0.18, *P* ═ 0.001), serum creatinine (*r* ═ 0.16, *P* ═ 0.003), triglyceride (*r* ═ 0.34, *P* < 0.001), and LDL-cholesterol (*r* ═ 0.2, *P* < 0.001).

In ROC analysis of the subgroup cohort, it was revealed that a CAR level higher than 1.22 had 78% sensitivity and 83% specificity in detecting DN (AUC 0.88, *P* < 0.001, 95% CI 0.84–0.92).

The subgroup cohort was also subjected to regression analysis taking into account CAR, age, gender, BMI, diabetes duration, waist circumference, and LDL cholesterol and it was found that CAR was an independent risk factor for DN since a unit increase in CAR increased the risk of DN by 25% (OR 0.252, *P* < 0.001, 95% CI 0.17–0.373).

## Discussion

The present study yielded several important results: (a) patients with DN exhibited higher CAR levels compared to diabetic patients without DN, (b) significant correlations were found between CAR and markers of poor diabetic outcome in patients with T2DM, (c) CAR demonstrated considerably high sensitivity and specificity in detecting DN, and (d) increased CAR levels were identified as an independent risk factor for DN (with a unit increase in CAR increasing the odds of DN by 34%).

In the present study, the BMI level of the DN group was found to be greater than that of the non-DN group. Similar findings have been reported in the literature, such as a study by Smith et al. [11], which involved 218 subjects and found that obesity was a risk factor for the development of DN. A more recent study by Callaghan et al. [12] reported that obesity was an important risk factor for DN in subjects with diabetes mellitus. Another report from Africa showed that patients with DN were more likely to have a higher BMI compared to diabetic subjects without neuropathy [13]. Additionally, other indices of obesity, such as waist circumference, whole body fat, and visceral fat mass, have been found to be related to neuropathy in diabetic patients [14]. Furthermore, the duration of diabetes mellitus was found to be another factor associated with DN in the present study. An Indian study including 336 diabetic subjects revealed that the duration of diabetes was associated with the presence of DN [15]. A recent meta-analysis involving more than 12,000 diabetic patients found that the duration of diabetes was significantly associated with an increased risk of diabetic polyneuropathy [16]. These findings suggest that increased disease duration is associated with an increased risk of neuropathy in the diabetic population, which is consistent with the results of the present study, where the duration of diabetes was significantly longer in the DN group compared to the non-DN group.

Fasting plasma glucose was identified as an important factor in the development of neuropathy in diabetic patients. Poor control of diabetes mellitus resulting in high blood glucose levels increases the risk of diabetic polyneuropathy. Conversely, enhancing blood glucose control could prevent the development of DN [17]. Fluctuations in blood glucose levels in diabetic subjects have negative effects on the development and progression of diabetic polyneuropathy, as suggested by a study by Oyibo et al. [18]. Moreover, not only diabetic subjects but also patients with prediabetes are at risk of neuropathy. The rate of impaired glucose tolerance has been reported to be increased in individuals with peripheral neuropathy. Additionally, metabolic syndrome rates were found to be increased in subjects with symmetric peripheral neuropathy [19]. Consistent with the literature, the present study reported higher fasting blood glucose levels in patients with DN compared to those without DN.

DN was found to be closely associated with HbA1c levels in patients with T2DM. Variability in glycated hemoglobin levels was suggested to be correlated with the presence of DN in the diabetic population [20]. Similarly, studies in different populations have linked DN to increased levels of HbA1c [21]. Moreover, extensive control of glucose by reducing HbA1c to target levels in a short interval results in the amelioration of neuropathy in diabetic subjects [22]. However, there are conflicting studies in the literature. A recent study by Casadei et al. [23] concluded that while HbA1c was a valuable marker of diabetes mellitus, its role in the diagnosis of DN should be elucidated further in subsequent studies with larger cohorts. This study, which found higher HbA1c levels in patients with DN than in patients without DN, supports previous studies in the literature indicating that high HbA1c increases the risk of DN.

Regarding serum creatinine levels, there was a slight but significant difference between the DN and non-DN groups in the present study, while the difference in eGFR was slightly insignificant. The CARE TIME study found an increased rate of DN in subjects with diabetic kidney disease, with higher levels of serum creatinine noted in those subjects [6]. Another study in the literature, which involved 100 subjects, revealed that the serum creatinine levels of patients with diabetic microvascular complications were significantly higher than those of diabetic subjects without microvascular complications [24]. Thus, our results were consistent with the data in the medical literature.

In addition, LDL-cholesterol levels were higher in the DN group than in the non-DN group in the present study, which aligns with similar findings in the literature. Tesfaye et al. [25] reported that elevated LDL-cholesterol was associated with an increased risk of DN in diabetic subjects. Subsequently, Andersen et al.’s [26] study confirmed these results by reporting a high risk of DN in diabetic patients with elevated LDL-cholesterol. Furthermore, a recent meta-analysis involving more than 30,000 subjects revealed that patients with high LDL-cholesterol were at an increased risk of DN [27]. However, conflicting reports exist in the literature about the role of LDL-cholesterol in DN. A Danish study in 2019 reported that increased LDL-cholesterol was not associated with a high risk of DN [28]. The results of the present study support the studies suggesting that high LDL-cholesterol increases the risk of DN.

Serum CRP is considered the prototype and most commonly used marker of inflammation. Serum albumin, on the other hand, is considered a negative acute-phase marker since it is reduced during infections and inflammatory conditions. The combined use of these two indices, CAR, has been studied in various conditions. The CARE TIME study previously reported that elevated CAR levels were associated with diabetic kidney injury [6]. The role of CAR has also been studied in malignant conditions. A recent study suggested that CAR was superior to other indices in predicting the outcome of subjects with colorectal carcinoma [29]. In addition, CAR was introduced as a prognostic indicator of survival in patients with non-small cell lung cancer [30]. Interestingly, a meta-analysis suggested that CAR was useful in predicting the outcome after treatment in patients with various types of cancer [31]. CAR has also been studied in rheumatologic conditions. In 2023, CAR was reported to have high sensitivity and specificity in predicting ANCA-associated vasculitis [32]. Recent studies have investigated its role in surgical procedures. For example, CAR was found to be useful in risk stratification of patients who received trans-catheter edge-to-edge mitral valve repair [33]. Moreover, higher CAR levels in patients undergoing surgery were associated with a higher rate of mechanical ventilation needed in the postoperative period [34]. Behera et al. [35] studied CAR levels in patients with pancreatitis and found that CAR was an independent predictor of mortality in those patients. All of these conditions were associated with an inflammatory burden, as is DN. Thus, our results showing increased CAR levels in DN appear to be compatible with the literature.

Inflammatory burden in certain conditions is reflected by serum CAR levels. A study from China revealed that increased CAR was a predictor of survival in patients with lung cancer [30]. Subsequently, CAR’s role in cancer has been confirmed by a meta-analysis [31]. Elevated CAR levels were proposed as a marker of inflammatory burden in diabetic nephropathy [6]. It was also reported to be linked with inflammation in rheumatological diseases [32]. In accordance with the literature data, our results indicated the diagnostic value of CAR in DN.

In a recent work by Bayrak [36], it has been reported that CAR was elevated in diabetic subjects with at least one diabetic complication compared to those without any diabetic complication. However, there was no correlation between CAR and any of the diabetic microvascular complications in that study [36]. In the present work, a similar increase in CAR in DN patients was noted. Moreover, CAR had considerably high sensitivity and specificity in detecting DN in diabetic subjects.

There are several limitations to the present study. The relatively small study population and retrospective design are the main limitations that may hinder the generalization of our results. Moreover, the single-center nature of the study is another limitation. However, to the best of our knowledge, this is the first study in the literature to report CAR as an independent risk factor for DN.

## Conclusion

In conclusion, elevated CAR levels could be considered as a marker of DN and warrant prompt evaluation in patients with T2DM. CAR can be a valuable additional diagnostic tool in this aspect due to its inexpensive and easy-to-assess nature.

## Data Availability

The data sets supporting the conclusions of this article and its supporting information are available from the corresponding author upon reasonable request.
